# Awake vs. Sedated Cannulation for Extra-Corporeal Membrane Oxygenation in Patients with COVID-19 Induced Acute Respiratory Distress Syndrome

**DOI:** 10.3390/jcm15020876

**Published:** 2026-01-21

**Authors:** Ori Galante, Anton Bukhin, Nitzan Sagie, Dekel Stavi, Yigal Kasiff, Yael Haviv, Maged Makhoul, Arie Soroksky, Meital Zikri-Ditch, Daniel Fink, Eduard Ilgiyaev

**Affiliations:** 1 Medical Intensive Care Unit, Soroka University Medical Center, Faculty of Health Sciences, Ben Gurion University of the Negev, Beer-Sheva 84101, Israel; 2 Medical Division, Soroka University Medical Center, Faculty of Health Sciences, Ben Gurion University of the Negev, Beer-Sheva 84101, Israel; antonbu3@gmail.com; 3 Clinical Research Center, Soroka University Medical Center, Faculty of Health Sciences, Ben Gurion University of the Negev, Beer-Sheva 84101, Israel; sagieni@post.bgu.ac.il; 4 Division of Anesthesia, Pain Management, and Intensive Care, Tel-Aviv Sourasky Medical Center—Ichilov Hospital, Faculty of Medicine, University of Tel Aviv, Tel Aviv 64239, Israel; dekel.stavi@gmail.com; 5 Department of Cardiothoracic Surgery, Sheba Medical Center, Ramat Gan 52621, Israel; yigal_k@bbalev.co.il; 6 General Intensive Care Unit, Sheba Medical Center, Ramat Gan 52621, Israel; yael.haviv@sheba.health.gov.il; 7 Department of Cardiothoracic Surgery, Rambam Health Care Campus, Haifa 31096, Israel; magedmakhoul@gmail.com; 8 Intensive Care Unit, Wolfson Medical Center, Sackler School of Medicine, University of Tel Aviv, Tel Aviv 64239, Israel; soroksky@gmail.com; 9 Intensive Care Unit, Kaplan Medical Center, Hebrew University, Jerusalem 99308, Israel; meital_zik@hotmail.com; 10 Shaare Zedek Medical Center, Hebrew University, Jerusalem 99308, Israel; dfink@szmc.org.il; 11 Intensive Care Unit, Shamir Medical Center, Sackler School of Medicine University of Tel Aviv, Tel Aviv 64239, Israel; eduard32@gmail.com

**Keywords:** extra-corporeal membrane oxygenation, COVID-19, respiratory distress syndrome, prognosis, respiration

## Abstract

**Background:** Veno-venous extra-corporeal membrane oxygenation (ECMO) cannulations are mostly performed while patients are heavily sedated and mechanically ventilated. For patients with acute respiratory distress syndrome (ARDS), cannulating for ECMO while awake and spontaneously breathing, as well as treating without sedation and mechanical ventilation, has potential advantages. This study aimed to compare clinical outcomes between patients cannulated for ECMO while awake and patients cannulated while sedated and mechanically ventilated. **Methods:** A retrospective multicenter study. Data were accessed from the Israeli ECMO registry of patients with COVID-19-induced ARDS treated at eight ECMO centers in Israel. The study group comprised 24 patients who were cannulated while awake and spontaneously breathing. A control group comprised 96 patients who were cannulated after sedation and mechanical ventilation, matched 1:4 by age, sex, and body mass index. The primary outcome was six-month survival. Secondary outcomes were: the duration of ECMO therapy, the duration of invasive mechanical ventilation-free ECMO therapy, and the duration of invasive mechanical ventilation. **Results**: The mean age was 52 + 11 years; 78% were males. Fifteen patients (63%) in the study group were eventually intubated. The mean durations on ECMO and in the intensive care unit did not differ between the groups. The study group had a higher six-month survival (75% vs. 49%, *p* = 0.02) and fewer infectious complications such as pneumonia or bacteremia (21% vs. 40%, *p* < 0.001) compared to the control group. After adjusting for PO_2_/FiO_2_ ratio and for the COVID-19 variant, the hazard ratio was 0.45 (C.I 0.19–1.06, *p* = 0.069). **Conclusions**: Awake VV-ECMO cannulation in COVID-19-induced ARDS is feasible in selected patients and was associated with higher survival in unadjusted analyses. However, after adjustment for key covariates, this association was attenuated and did not reach statistical significance.

## 1. Introduction

The use of veno-venous extra-corporeal membrane oxygenation (VV-ECMO) to treat patients with severe acute respiratory distress syndrome (ARDS) has increased since the 2009 H1N1 influenza pandemic and peaked during the COVID-19 pandemic [1]. Most VV-ECMO cannulations are performed while patients are heavily sedated and mechanically ventilated. The awake ECMO strategy, i.e., cannulating patients with ARDS for ECMO while awake and spontaneously breathing, as well as treating them without sedation and mechanical ventilation, has several potential advantages. These include reducing ventilatory-induced lung injury and ventilatory-associated pneumonia, as well as increasing functional residual capacity [2]. Moreover, by contracting the diaphragm, patients who breathe spontaneously achieve more balanced ventilation–perfusion matching [2] and avoid ventilatory-induced diaphragmatic damage [3]. Patients treated with ECMO while awake can undergo physiotherapy and rehabilitation much earlier, during the peak of their disease severity. At this disease stage, patients treated conservatively are in deep sedation and even under muscle relaxants. Awake patients can communicate and potentially have a reduced risk of delirium [4].

Currently, there is a notable absence of large-scale studies of patients treated with ECMO that directly compared outcomes between those cannulated while awake and while sedated. Though individual case reports and small-scale studies have provided insights into the potential benefits of awake ECMO [5,6,7,8,9,10], a comprehensive comparison of outcomes between these two approaches is lacking in the existing literature.

We recently published our experience in awake ECMO cannulation of patients with ARDS [4]. We demonstrated, as a proof of concept, the feasibility of treating patients with severe ARDS using VV-ECMO without sedation and mechanical ventilation in selected patients.

In the current study, we collected and analyzed data from all the Israeli intensive care units that practiced awake ECMO in patients with COVID-19 ARDS. We compared the outcomes of patients treated by awake versus matched mechanically ventilated ECMO.

## 2. Materials and Methods

### 2.1. Study Design and Population

We conducted a multicenter, retrospective cohort study in eight ECMO centers in Israel. Data were gathered from the Israeli ECMO registry of patients with COVID-19-induced ARDS who were treated with ECMO. The Israeli ECMO Society and ECMO registry were founded in 2019 and include 13 medical centers that operate ECMO.

We included patients with confirmed COVID-19 via polymerase chain reaction, who were treated with VV-ECMO for COVID-19-induced ARDS from April 2020 to December 2022. Patients were excluded if they were under 18 years of age or if their records indicated poor data collection.

The study group was defined as patients who were cannulated for ECMO while awake and spontaneously breathing. The control group was established by using propensity score matching in a 1:4 ratio, by age, sex, and body mass index, of patients who were cannulated for ECMO after sedation and mechanical ventilation.

Decisions to initiate ECMO in sedated and mechanically ventilated patients were based on the judgment of the treating physician according to the Extra-Corporeal Life Support Organization (ELSO) guidelines and EOLIA criteria [11,12].

Notably, specific guidelines and criteria have not been issued regarding the selection of patients for whom to attempt awake ECMO. During the study period, diagnoses and clinical decisions, including the decision to initiate ECMO, were made at each of the included centers according to the judgment of the treating team. For patients deemed to require mechanical ventilation with a high likelihood of eventually requiring ECMO, decisions were made as to whether to intubate or attempt awake ECMO. Generally, when patients were alert enough and able to collaborate, awake ECMO was considered an alternative to mechanical ventilation [4]. 

The data collected included demographic information, comorbidities, and pre-ECMO baseline parameters, including respiratory support modality, gas exchange data, and the duration of invasive mechanical ventilation when it was used. ECMO-related parameters during ECMO therapy were also recorded: total ECMO duration, maximal blood flow, maximal gas flow, and ECMO-related complications.

In order to compare the severity of respiratory failure before ECMO initiation, we measured arterial blood gas immediately before ECMO cannulation, while patients were breathing 100% oxygen; hence, in intubated patients, these values represents PO_2_/FiO_2_ ratio, and in non-intubated patients, a proximity of PO_2_/FiO_2_ ratio.

We also calculated the value of Alveolar-arterial (A-a) gradient, although, like the PO_2_/FiO_2_ ratio, A-a gradient is also limited in non-ventilated patients.

### 2.2. Definitions

ARDS was defined according to the “Berlin criteria.” [13].

Thrombotic events were defined as either venous or arterial thrombosis, heparin-induced thrombocytopenia, or ECMO circuit thrombosis necessitating circuit change. Bleeding was defined as bleeding necessitating treatment with blood products. Secondary infections were defined as pneumonia, bacteremia, or any other non-COVID-19-induced sepsis.

### 2.3. Outcomes

The primary outcome was mortality during six months of follow-up. Secondary outcomes were as follows: mortality on ECMO, the duration of ECMO therapy, the duration of invasive mechanical ventilation-free ECMO therapy, which was calculated as the number of days on ECMO in which the patient was not intubated and was spontaneously breathing, and the duration of invasive mechanical ventilation.

### 2.4. Statistical Analysis

Baseline characteristics of the study groups were summarized as percentages for categorical variables and as means with standard deviations for continuous variables. Comparisons of baseline characteristics between groups were performed using the chi-square test or Fisher’s exact test, as appropriate, for categorical variables, and the Student’s *t* test or Wilcoxon rank-sum test for continuous variables, depending on data distribution. Covariate balance between groups after matching was evaluated using standardized mean differences, with values less than 0.1 considered indicative of adequate balance. Overall survival was estimated using the Kaplan–Meier method and compared between groups using the log-rank test. Survival analyses were further conducted using Cox proportional hazards regression to estimate hazard ratios (HRs) with corresponding 95% confidence intervals (CIs) for all-cause mortality during a six-month follow-up period, comparing patients who were awake versus sedated at the time of ECMO cannulation. COVID-19 variants and P/F ratio were included in the models to adjust for potential confounding effects. All statistical tests were two-sided, and *p*-values <0.05 were considered statistically significant. Statistical analyses were performed using R software (version 4.3.1; R Foundation for Statistical Computing, Vienna, Austria).

## 3. Results

We identified 24 patients who were cannulated for ECMO during the study period, while awake and spontaneously breathing, and matched them to 96 controls. Table 1 shows baseline characteristics of the study population. The mean age was 52 years (standard deviation [SD] 11), and 78% were males. The mean body mass index (BMI) was 32 kg/m^2^ (SD 8). Among those who were cannulated for ECMO while awake (the study group) compared to those who were cannulated while sedated (the control group), the mean Sequential Organ Failure Assessment (SOFA) score was lower (5.2 vs. 9.1, *p* < 0.001). Compared to the control group, the study group’s pre-ECMO PO_2_ (while breathing 100% oxygen) and PCO_2_ were lower: 53 mmHg vs. 61 mmHg (*p* = 0.052) and 43 mmHg vs. 65 mmHg (*p* < 0.001), respectively. Twelve (50%) of the patients in the study group were supported with High-Flow Nasal Cannula before and during cannulation, and ten patients (41.7%) were supported with non-invasive ventilation. After cannulation, all patients in the study group received High-Flow Nasal Cannula. Although the dominant COVID-19 variant shifted during the study period (from wild type to Alpha, Delta, and Omicron), variant distribution did not differ significantly between groups (*p* = 0.1), nor did the mean ECMO blood flow differ between the groups.

Fifteen out of the twenty-four patients (63%) who were cannulated for ECMO while awake eventually required intubation and mechanical ventilation during the ECMO run. Intubation occurred after a mean of 12 (SD 12) days from cannulation. The mean mechanical ventilation-free days in the study group was 12.3 (SD 15). Intubation was required most commonly due to respiratory distress with inadequate control of respiratory drive (n = 10). Other indications included sepsis (n = 3), clinically significant bleeding (n = 1), and unplanned cannula dislodgement (n = 1).

**Figure 1:**
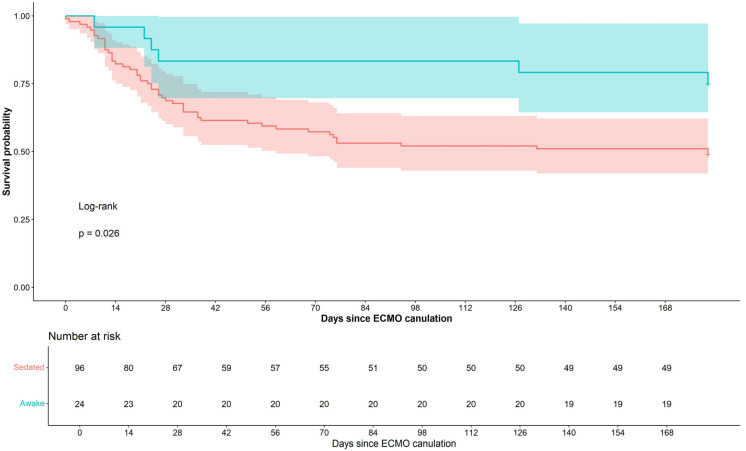
Kaplan-Meier plot showing survival of patients cannulated for ECMO while sedated and while awake.

Table 2 compares the outcomes of the study and control groups. Durations of time on ECMO and in the intensive care unit did not differ significantly between the groups. Six-month survival was higher in the study compared to the control group (75% vs. 49%, *p* = 0.02). Figure 1 shows the Kaplan–Meier plots of the two groups.

Table 3 compares the complication rate of the study and control group. Rates of mechanical and infectious complications were lower in the study compared to the control group: 21% vs. 67% (*p* < 0.001) and 25% vs. 44% (*p* = 0.078), respectively.

None of the patients in the study group developed pneumonia, compared to 44.8% of the patients in the control group. Among the subgroup of patients who were cannulated while awake and never required ventilation, no infectious complications occurred during the entire ECMO run. Hemostatic complications, either thrombotic or hemorrhagic, occurred more frequently in the study than the control group (54% vs. 29%, *p* = 0.021).

Table 4 presents the results of the COX regression model: The HR for six-month mortality was 0.40 for the study group compared to the control group (95% CI 0.17–0.92, *p* = 0.032). Adjustment for PO_2_/FiO_2_ ratio yielded a HR of 0.41 (95% CI 0.17–0.97, *p* = 0.043). Adjustment for the COVID-19 variant yielded a HR of 0.44, (95% CI 0.19–1.03; *p* = 0.059).

## 4. Discussion

Our study failed to show a statistically significant association between awake VV-ECMO cannulation and improved clinical outcomes in patients with COVID-19-induced ARDS, compared with cannulation under sedation and mechanical ventilation. A number of studies reported better outcomes of awake compared to sedated ECMO in patients with respiratory failure awaiting lung transplantation [14,15,16]. While those patients and patients with chronic obstructive pulmonary disease have reasonable toleration for being awake on ECMO, for patients with ARDS, respiratory drive control is more difficult to achieve. This is consequent to their lung pathophysiology, which includes severe inflammation, large parenchymal consolidation, and low lung compliance. Hence, among awake patients with ARDS, intubation is more common [17]. Despite these challenges, several case series have shown the feasibility of an awake ECMO strategy in patients with ARDS [4,18,19,20,21], though evidence of the actual benefit of this strategy is scarce. Although 63% of our awake ECMO patients were eventually intubated, they had a mean of 12.3 (SD 15) mechanical ventilation-free days. The primary indication for intubation was the inability to control respiratory drive associated with worsening respiratory distress. Notably, no cases of pneumonia were observed among patients cannulated while awake, compared with 44.8% in the control group. In addition, none of the patients who remained non-intubated throughout their ICU admission developed infectious complications. Although causality cannot be inferred, these observations suggest a potential benefit of avoiding endotracheal intubation in selected patients with ARDS.

Awake veno-arterial-ECMO cannulation was also found to be associated with improved mortality [22]. Mohamed et al. analyzed data of 28,627 patients from the ELSO registry and compared the outcomes of 797 patients who were cannulated for VV-ECMO while awake and spontaneously breathing to those who were cannulated while on mechanical ventilation [23]. Among the patients who underwent awake cannulation, the mean age was older, and the prevalence of chronic lung diseases and ischemic heart disease was greater. Approximately 35 percent of them were cannulated with the intent of pursuing a lung transplant, compared to only 4.7 percent in the mechanically ventilated group. Survival to hospital discharge did not differ significantly between the groups. The population of that study was diverse and included patients who were cannulated for VV-ECMO due to various lung pathologies, for which disease course and prognosis vary widely. Because of the substantial diversity in case-mix and underlying etiologies, in the study by Mohamed et al. [23], the ability to draw specific conclusions regarding the clinical utility of awake VV-ECMO cannulation is limited. Our study, in contrast, includes only patients with COVID-19-induced ARDS, and therefore, different outcomes may be expected in this more homogeneous population.

This may explain the differences in outcomes between the two studies.

Comparison of ARDS severity between the study and control groups is challenging, given that many commonly used severity parameters, such as arterial blood gas indices and the SOFA score, are strongly influenced by ventilatory status and support settings. We report a lower pre-ECMO PO_2_ (while breathing 100% oxygen) and PCO_2_ in the study group compared to the control group. In their large-scale study, Mohamed et al. also found lower PCO_2_ in the awake group, though the PO_2_ was higher [23]. As PO_2_ was actually lower in the study group, one may argue that it may reflect a more severe disease. We assume that our findings of differences in blood gas results between the study and control groups do not reflect a difference in disease severity, but merely differences resulting from the ventilation status (i.e., spontaneous vs. mechanical ventilation). In patients on mechanical ventilation, the use of lung protective ventilation and high positive end expiratory pressure may result in permissive hypercapnea and higher PO_2_. After adjustment for the PaO_2_/FiO_2_ ratio, the hazard ratio for mortality remained significantly lower in the study group (HR 0.41; 95% CI 0.17–0.97; *p* = 0.043). Given the temporal shift in dominant COVID-19 variants during the study period and their differing disease profiles, we further adjusted the model for COVID-19 variants. Following this additional adjustment, the hazard ratio remained low, although the association did not reach statistical significance. We found a lower mean SOFA score among those cannulated while awake compared to the control group (5.2 vs. 9.1, *p* < 0.001). Mechanical ventilation status and the level of consciousness are major parameters in the SOFA scoring. Sedation and mechanical ventilation, which may also induce hypotension and the need to use vasopressors, add an average of 4 points to the SOFA score, so by definition, the SOFA score differed between the two groups.

This study has several strengths. It is the largest study to date to focus on the prognosis of awake ECMO cannulation, specifically in patients with viral ARDS. Patients were included from eight hospitals that practice an awake ECMO cannulation strategy. This study has several limitations. First, the relatively small number of patients who were cannulated while awake may have led to statistical error. Second, the retrospective design introduces the possibility of selection bias, as clinicians may have preferentially selected patients with more favorable clinical profiles for awake cannulation. Awake ECMO cannulation was not randomly assigned and depended heavily on clinician judgment, patient cooperation, preserved consciousness, and perceived physiologic stability. These same characteristics may associate with improved prognosis and therefore introduce substantial confounding by indication. In particular, a phenomenon analogous to “depletion of susceptibility” is likely present. Patients with more severe early trajectories may have preferentially intubated and cannulated under sedation, thereby enriching the control group with patients at higher baseline risk of death while depleting the awake group of such individuals. This mechanism can create a spurious appearance of benefit in the intervention group even in the absence of a true treatment effect. Although this could have contributed to the observed outcome differences, no significant differences were found in baseline medical characteristics or disease severity. In addition, ICU length of stay and ECMO duration were comparable between groups, whereas shorter durations would be anticipated in patients with milder disease.

As randomized controlled trials in this population are extremely challenging due to the rarity of the disease, center-level heterogeneity, and expertise requirements. High-quality, larger-scale observational studies using target trial emulation may represent the most feasible path to further investigate the association between awake ECMO cannulation and prognosis in ARDS patients.

## 5. Conclusions

Awake VV-ECMO cannulation in COVID-19-induced ARDS is feasible in selected patients. Although unadjusted analyses suggested an association with improved survival and fewer complications, this association was attenuated after multivariate adjustment. These findings should be considered hypothesis-generating and support the need for larger studies.

## Figures and Tables

**Table 1:** Baseline characteristics of patients cannulated for ECMO while sedated and while awake.

Characteristic	Overall, N = 120	Sedated, N = 96	Awake, N = 24	*p*-Value
Age, years, Mean (SD)	51.7 (11.1)	51.6 (10.9)	52.3 (11.8)	0.89
Male sex, N (%)	94 (78.3)	75 (78.1)	19 (79.2)	0.91
BMI, kg/m^2^, Mean (SD)	32.0 (7.5)	32.1 (7.5)	31.6 (7.9)	0.79
Smoker, N (%)	8 (6.7)	5 (5.2)	3 (12.5)	0.20
Diabetes, N (%)	28 (23.3)	22 (22.9)	6 (25.0)	0.83
Cardiovascular disease, N (%)	12 (10.0)	9 (9.4)	3 (12.5)	0.70
Pre-ECMO HFNC, N (%)	12 (10.0)	0 (0.0)	12 (50.0)	<0.001
Pre-ECMO NIV, N (%)	10 (8.3)	0 (0.0)	10 (41.7)	<0.001
Pre-ECMO Prone positioning, N (%)	44 (36.7)	44 (45.8)	0 (0.0)	<0.001
Pre-ECMO Nitric oxide, N (%)	72 (60.0)	65 (67.7)	7 (29.2)	<0.001
Pre-ECMO PO_2_ (while breathing 100% O_2_) Mean (SD)	59.8 (19.5)	61.0 (19.8)	53.0 (11.8)	0.052
Pre-ECMO PCO_2_, Mean (SD)	58.9 (21.2)	65.0 (21.3)	43.0 (9.4)	<0.001
Pre-ECMO pH, Mean (SD)	7.31 (0.12)	7.28 (0.12)	7.39 (0.07)	<0.001
Pre-ECMO A-a gradient, Mean (SD)	578(35.7)	567 (34.8)	606 (18.9)	<0.001
SOFA, Mean (SD)	8.3 (3.4)	9.1 (3.1)	5.2 (2.2)	<0.001
COVID-19 variant, N (%)				0.10
Wild	28 (23.3)	26 (27.1)	2 (8.3)	
Alpha	45 (37.5)	36 (37.5)	9 (37.5)	
Delta	39 (32.5)	27 (28.1)	12 (50.0)	
Omicron	8 (6.7)	7 (7.3)	1 (4.2)	
ECMO blood flow, Mean (SD)	4.7 (0.9)	4.8 (0.9)	4.7 (0.9)	0.78

BMI—Body Mass Index, ECMO—Extra-Corporeal Membrane Oxygenation, HFNC—High-Flow Nasal Cannula, NIV—Non-Invasive Ventilation, SOFA—Sequential, Organ, Assessment Score.

**Table 2:** Comparison of clinical outcomes between patients cannulated while sedated and while awake.

Characteristic	Overall, N = 120	Sedated, N = 96	Awake, N = 24	*p*-Value
LOS in ICU (d), Mean (SD)	39.9 (32.7)	37.9 (30.2)	46.8 (39.9)	0.65
LOS in ICU (d) in Survivors, Mean (SD)	42.1 (32.9)	40.6 (30.3)	45.8 (38.7)	0.82
ECMO duration (d), Mean (SD)	27.8 (26.5)	25.5 (23.1)	37.1 (36.3)	0.32
ECMO duration (d) in survivors, Mean (SD)	27.0 (24.3)	24.1 (19.6)	34.9 (33.4)	0.39
Ventilation, N (%)	111 (92.5)	96 (100)	15 (62.5)	<0.001
Ventilation-free days, Mean (SD)	2.0 (8.3)	0.0 (0.0)	12.0 (15.0)	<0.001
Survived to ECMO decannulation, n (%)	78 (65.0)	57 (59.3)	21 (87.5)	0.011
Survived to ICU discharge, N (%)	67 (55.8)	49 (51.6)	18 (75.0)	0.039
Six-month survival, N (%)	65 (54.2)	47 (49.0)	18 (75.0)	0.022

LOS—Length of Stay, ICU—Intensive Care Unit, ECMO—Extra-Corporeal Membrane Oxygenation.

**Table 3:** Comparison of the complication rate between patients cannulated while sedated and while awake.

Characteristic	Overall, N = 120	Sedated, N = 96	Awake, N = 24	*p*-Value
Hemostatic complications, N (%)	42 (35.0)	34 (35.4)	8 (33.3)	0.85
Thrombotic	24 (20.0)	18 (18.8)	6 (25.0)	0.57
Bleeding	34 (28.3)	27 (28.1)	7 (29.2)	0.92
Infectious complications, N (%)	49 (40.8)	43 (44.8)	6 (25.0)	0.078
Pneumonia	43 (35.8)	43 (44.8)	0 (0.0)	<0.001
Sepsis	27 (22.5)	21 (21.9)	6 (25.0)	0.74
Mechanical complications, N (%)	69 (58)	64 (67)	5 (21)	<0.001

**Table 4:** Results of COX regression models.

Model	HR (95% CI) ^1^	*p*-Value
Awake group	0.40 (0.17 to 0.92)	0.032
Awake group + P/F ratio	0.41 (0.17 to 0.97)	0.043
Awake group + COVID-19 variant	0.44 (0.19 to 1.03)	0.059
Awake group + P/F ratio + COVID-19 variant	0.45 (0.19 to 1.06)	0.069

^1^ HR = Hazard Ratio, CI = Confidence Interval.

## Data Availability

Deidentified tabulated data can be provided upon reasonable request, approved by the Israel National Ethics Committee.

## Abbreviations

The following abbreviations are used in this manuscript:

ARDS	Acute Respiratory Distress Syndrome
BMI	body mass index
ECMO	extra-corporeal membrane oxygenation
LOS	Length of Stay
ICU	Intensive Care Unit
HR	hazard ratio
OR	Odds Ratio
SOFA	Sequential Organ Failure Assessment
VV	veno-venous
